# Revisiting the Micropipetting Techniques in Biomedical Sciences: A Fundamental Prerequisite in Good Laboratory Practice

**DOI:** 10.6026/97320630016008

**Published:** 2020-01-01

**Authors:** Peter Natesan Pushparaj

**Affiliations:** 1 Center of Excellence in Genomic Medicine Research, King Abdulaziz University, Jeddah-21589, Kingdom of Saudi Arabia; 2 Department of Medical Laboratory Technology, Faculty of Applied Medical Sciences, King Abdulaziz University, Jeddah-21589, Kingdom of Saudi Arabia

## Abstract

The underlying technical and operational knowledge of micro-pipetting is essential for scientists, technicians, and students to achieve precise results from their experiments in Biomedical Sciences and other related disciplines. Since it is one of the crucial prerequisites for Good Laboratory Practices (GLPs), the key fundamentals of the micro-pipetting methods, such as forward and reverse pipetting and the importance of ergonomics and maintenance etc., are revisited in this Editorial report.

## Background

**Figure 1:**
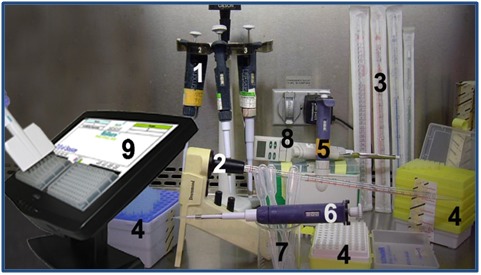
Various Pipettes and Related Items: 1) Adjustable pipettes, from left: P-20 to P-200 (20-200 µl), P-2 to P-20 (2-20 µl), P-100 to P-1000 (100-1000 µl); 2) Graded transfer pipette and electric pipette filler; 3) 25 mL, 10 mL, 5 mL, and 2 mL transfer pipettes; 4) Disposable tips for adjustable pipettes; 5) 12-channel adjustable pipette for microplates; 6) Low-retention 0.5-10 µl adjustable pipette; 7) Squeezable transfer pipettes; 8) Digital adjustable pipette; 9) Light-guided pipetting system.(Courtesy of Steinsky at English Wikipedia (https://commons.wikimedia.org/wiki/ File:Pipettes.jpg), "Pipettes, "marked as public domain, more details on Wikimedia Commons: https://commons.wikimedia.org/wiki/Template:PD-user)

Biomedical researchers strive to achieve optimal and reliable results from experiments either in diagnostics or research and development (R and D) in the laboratories [1,2]. In order to achieve this objective, basic knowledge about the fundamental steps involved in micropipetting techniques is one of the essential prerequisites [3-5]. There are many types of air and positive displacement micropipettes available commercially, such as singlechannel, multi-channel, manual or electronic, etc. with several accessories to perform experiments in Biomedical Sciences and related disciplines [6-8] ( Figure 1).

**Figure 2:**
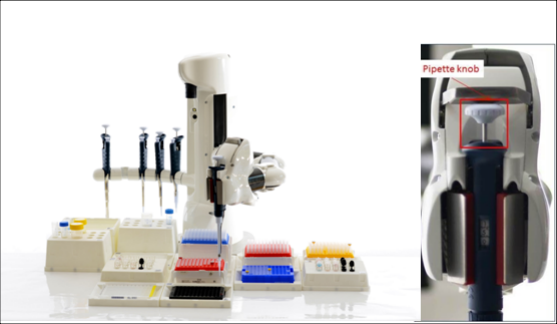
Liquid Handling Robot. (A) Forward and Reverse Pipetting can be performed using a Liquid Handling Robot capable of using a manual pipette in the Biomedical Laboratories (Courtesy of Pzucchel and shared based on CC BY-SA 3.0 license) (B) Robotic Hand in the Liquid Handling System holding a Gilson Pipette (Courtesy of Pocar19 and shared based on CC BY-SA 3.0 license)

Several companies sell manual and electronic micropipettes,automated liquid handling robots (Figure 2), and liquid handling workstations. Therefore, the technical information about each type of micropipette, as well as other accessory systems and its operational protocols, should be thoroughly explored before use in the laboratory [6-8].

## Basic micropipetting techniques in Biomedical Sciences:

**Figure 3:**
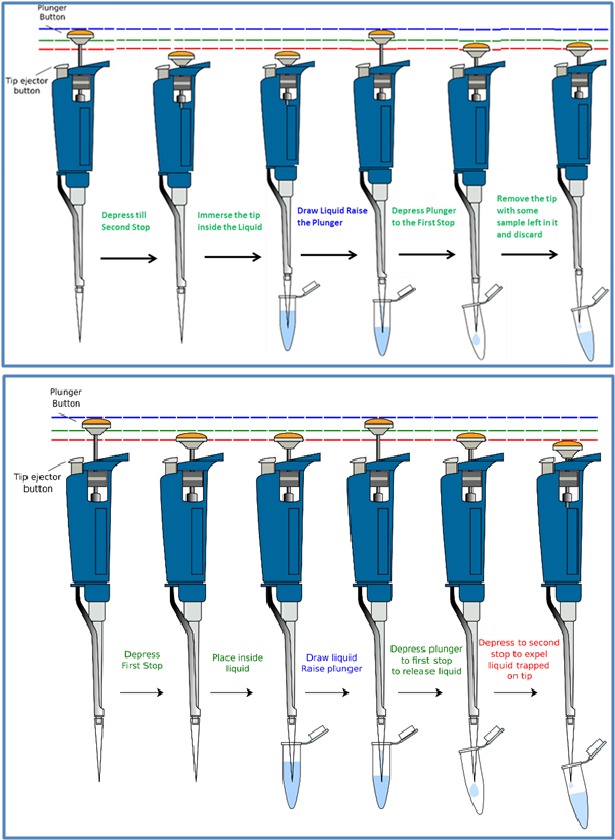
(A) In forward pipetting, press the plunger button slowly to the first stop outside of the solution to displace air and avoid blowing bubbles into it. Cautiously draw the plunger up gradually and follow the liquid to prevent drawing air. Press the plunger in the destination tube until the second stop to expel full volume from the tip (Courtesy Biology Open Educational Resources (Bio-OER), Open Lab at City Tech CUNY, USA and shared based on CC BYNC- SA 4.0). (B) In reverse pipetting, depress the plunger button slowly to the second stop outside of the solution to displace air and avoid blowing bubbles into it. Cautiously draw the plunger up gradually and follow the liquid to prevent drawing air. Press the plunger in the destination tube until the first stop to expel the sample and discard the tip with the remaining solution in the waste container (Modified based on the source from Biology Open Educational Resources (Bio-OER), Open Lab at City Tech CUNY, USA and shared alike based on CC BY-NC-SA 4.0)

Basically, the micropipetting techniques are classified into two main types, namely, Forward Pipetting and Reverse Pipetting [6-8].The first step in micropipetting will be to adjust the micrometer in the pipette to set the volume required based on the assay protocol.In the second step, an appropriate disposable tip will be attached by gently pushing the pipette shaft over a tip, and the plunger button will slowly be pressed to the first stop outside of the solution to displace air and avoid blowing bubbles into it.Cautiously the plunger will be drawn up gradually to prevent drawing air into the pipette. Finally, the plunger will be pressed to release the reagent or solution in the destination tube until the second stop to expel full volume from the tip. This micro-pipetting method is called as Forward Pipetting [4-8] (figure 3A).

Conversely, in the reverse pipetting method, after adjusting the micrometer to the desired setting, the plunger button will be depressed slowly to the second stop outside of the solution to displace air and avoid blowing bubbles into it. Cautiously, the plunger will then be drawn up gradually and follow the liquid to prevent drawing air. Finally, the plunger will be depressed in the destination tube until the first stop to expel the sample, and the tip will be discarded with the remaining solution in the appropriate waste container [4-8] (figure 3B). However, there are several key technical and environmental factors that influence the precision and accuracy of both forward and reverse pipetting methods [6-8].

## Importance of immersion angle and immersion depth in micropipetting:

To improve the accuracy, immerse the tip into the liquid, just below the meniscus at a vertical angle (∼90 degrees), and avoid aspirating air into the tip. Approaching the liquid between the angles from 45 to 60 degrees will significantly affect the accuracy of the pipetting.Immerse the pipette tip just below the meniscus and avoid immersing the tip either too deep or too shallow. Immersing too deep may increase the aspiration volume, whereas positioning the tip too near to the liquid surface can lead to the aspiration of air into the pipette resulting in inaccurate volume [4-8].

## Importance of Pre-rinsing, Speed, and Rhythm, and Dispensing:

Pre-rinsing the pipette tips for few times helps to neutralize capillary effects and balances the air temperature inside the tip with that of the sample. Pre-rinsing dramatically improves the accuracy of pipetting, especially very small micro-volumes. The rhythm and speed of pipetting should be consistent, and it will significantly improve pipetting accuracy. Fast or "erratic" aspiration may lead to aerosols, contamination of the pipette shaft, splashing of liquid, and loss of efficiency and sample volume. During dispensing, you should touch the vessel wall with the tip at an angle of 45 degrees to release the sample, and then slide it up the wall to prevent liquid from clinging to the orifice. However, the aqueous (non-viscous) liquids can also be dispensed either onto the surface or into the liquid [4-8].

## Environmental and technical factors influencing the micro pipetting:

The appropriate temperature for micropipetting is 21.5°C (±1°C),and the samples should be at the same temperature as the room in which you're working. To achieve this, you need to keep the samples to equilibrate for 20 minutes. Any significant or sudden temperature changes to the samples or micropipettes might reduce accuracy. Pipetting at a constant temperature improves the accuracy and precision of pipetting. Importantly, the dissipation of heat from the hand during long pipetting sessions may cause the expansion of air inside the pipette and lead to inaccurate results.This can be avoided by keeping the pipette on its stand between pipetting sessions, instead of holding it in your hand. Users should refrain from setting a pipette's micrometer to less than 10% of its maximum volume and change appropriate pipettes to aspirate and dispense lower sample volumes [4-8].

## The importance of ergonomics in micropipetting:

Following good ergonomic practices, augments accuracy and performance. Hand and body fatigue may cause errors, especially when dispensing small quantities and large numbers of samples.Fatigue and any injury can be avoided by good posture,finger hooks to relax the grip, taking a break for 3 to 5 minutes between long pipetting sessions, adjustable chair or stool to reach the tip boxes, sample tubes, and waste containers effortlessly, and organizing the worktable properly before the pipetting sessions [4-8].

## Importance of maintenance and periodic micropipette service:

The regular maintenance of micropipettes is essential to maintain precision and consistency in the results. The pipette is disassembled and cleaned, followed by calibration. The calibration is essential after cleaning the micropipettes. Some chemicals, such as organic solvents, affect certain parts of the pipette. It is essential to check and lubricate the O-rings, piston, and piston springs every week if the micropipette is used for regular pipetting of organic solvents,acids, and alkalis. The filter tips can be used to protect the micropipettes from sample contamination by preventing the entry of aerosols, excess liquids or foreign substances or particles [4-8].

## Conclusions and future directions:

The micropipetting skills are essential for achieving precise experimental results in all the areas of Biomedical Sciences.Furthermore, comprehensive knowledge about the micropipettes and its operational procedures is crucial for the users to accomplish optimal laboratory results. Importantly, researchers, technicians, and students should get proper practical training in micropipetting techniques as well as thorough orientation about the operational and technical instructions about the micropipettes available for experiments before embarking their laboratory activities.
